# Advantages of robotic assistance over a manual approach in simulated subretinal injections and its relevance for gene therapy

**DOI:** 10.1038/s41434-021-00262-w

**Published:** 2021-05-17

**Authors:** Reza Ladha, Thijs Meenink, Jorrit Smit, Marc D. de Smet

**Affiliations:** 1 Department of Ophthalmology, CHU St-Pierre and CHU Brugmann, Université Libre de Bruxelles, Bruxelles, Belgium; 2 Preceyes BV, Eindhoven, the Netherlands; 3 Department of Ophthalmology, Leiden University, Leiden, the Netherlands; 4 MIOS sa, Lausanne, Switzerland

**Keywords:** Neurological disorders, Gene therapy

## Abstract

Subretinal injection is a method for gene delivery to treat genetic diseases of the photoreceptors and retinal pigment epithelium. A reflux-free subretinal injection is important to allow effective, safe, and cost-effective gene therapy to the retina. We report on a comparison between manual and robotic assistance in simulated subretinal injections using an artificial retina model. Nine surgeons carried out the procedure with and without the Preceyes Surgical System, using an OPMI Lumera 700 Zeiss surgical microscope equipped with intra-operative optical coherence tomography. Success in creating a bleb without reflux, injection duration, drift, tremor, and increase in the diameter of the puncture hole were analyzed. Robotic assistance improved drift (median 16 vs 212 µm), tremor (median 1 vs 18 µm), enlargement of the retinal hole, and allowed for prolonged injection times (median 52 vs 29 sec). Robotic assistance allowed higher rate of bleb formation (8/9 vs 4/9 attempts) with a moderate reduction in reflux (7/9 vs 8/9 attempts) in this artificial model. Robotic assistance can significantly contribute to subretinal injections and provide quantifiable parameters in assessing surgical and clinical success of novel retinal gene therapies.

## Introduction

A subretinal injection (SI) is a method often proposed to treat genetic diseases affecting the photoreceptors and retinal pigment epithelium (RPE) [1, 2]. Under ideal conditions, SI should result in the placement of the entire therapeutic solution in the subretinal space in the immediate proximity of photoreceptors and RPE cells. This surgical approach is being used in on-going trials and was chosen for voretigene neparvovec-rzyl (Luxturna, Spark Therapeutics), the first gene therapy commercially available for a retinal genetic disease caused by biallelic mutations in RPE65 [2, 3].

While considerable effort has been deployed to optimize the vector’s design [4], to meet GMP standards [5], and define optimal patients for the procedure, several issues relating to delivery remain [1, 6, 7]. Reflux into the vitreous cavity is frequent where persistence of the virus can lead to immune response [8]. While a protocol has been defined for the delivery of gene product into the subretinal space, the outcome still depends on numerous uncontrolled factors: past surgical skill, successful completion of a learning curve [9], other members on the team [10]. Even then, several questions remain regarding execution of the procedure. Effective and safe delivery implies injecting an accurate quantity of the therapeutic agent into the subretinal space while minimizing any reflux into the vitreous cavity, overstretching of the retina, or penetrating deeper into the choroid with exposure of the viral vector to the systemic circulation. Related complications may be the development of a macular hole, epiretinal proliferation, a retinal detachment, subretinal and/or vitreous hemorrhages or the development of inflammation either immediate or delayed [11].

Physiologic hand tremor and visual depth resolution are two major limitations for eye surgeons while carrying out high precision tasks [12]. Tremor in dynamic tasks recorded during retinal surgery averages around 100 µm at the tip of an instrument [13]. In static positioning, micro jerks can push the instrument off target by 250 µm or more [14]. Limited depth perception through a microscope decreases the surgeon’s ability to perform precision task [15]. Taken together, these observations indicate that surgery aimed at the subretinal space is literally at the limit of human physiology.

Technological innovations can assist surgeons in overcoming physiological limitations, using robotics, precision micromanipulators, and live intraoperative optical coherence tomography (iOCT) [12]. Of particular interest to eye surgeons, the Preceyes Surgical System (PSS; Preceyes BV, Eindhoven, The Netherlands) is currently the only robotic-based technology dedicated to ophthalmology that has reached a commercial stage of development. It has been safely and successfully used in humans to peel epiretinal membranes and to subretinally inject recombinant tissue plasminogen activator (rtPA) in patients with subretinal hemorrhages [16]. PSS is a telemanipulation robotic system designed to filter out a surgeon’s tremor and to scale, limit or positionally assist movements of intraocular instruments. This enhances tool-tip positional stability and allows for a placement accuracy of 10 microns or less [14, 17]. A combination with high precision imaging provided by intraoperative optical coherence tomography (iOCT) devices or instrument-based OCT systems further enhances the ability to target retinal tissue with precision [18].

While the requirements for SIs using a robotic-based system are known, an assessment of human performance with and without robotic enhancement has not yet been reported [17–19].

In the present paper, we used a state-of-the-art surgical ophthalmic microscope to compare manual and PSS-assisted injections in a newly developed artificial retina model.

## Materials and methods

### Retina model

**Fig. 1:**
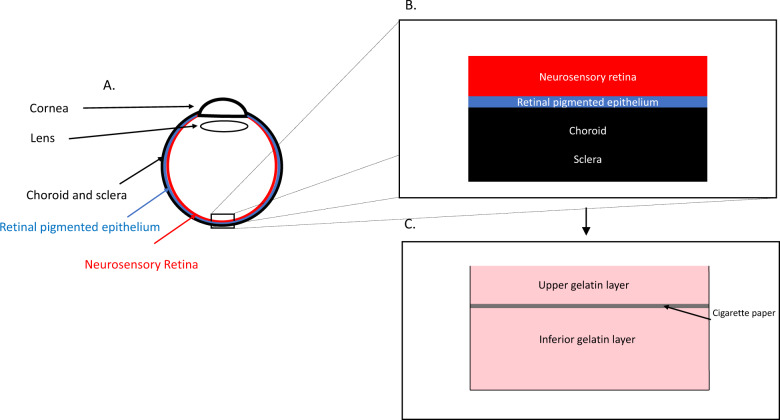
Schematics of the eye and gelatin model used in the experimental set-up. **a** A schematic representation of an eye. **b** A schematic section of the retina layers simulated by the model. **c** A schematic of the gelatin model.

The artificial retina consisted in two layers of pink gelatin, separated by a thin white layer of cigarette paper, deposited on standard microscope glass slide. The upper gelatin layer (~250 µm) simulates the neurosensory retina while the inferior gelatin layer (~500 µm) corresponds to the retinal pigmented epithelial layer, choroid, and sclera. Gelatin (Dr Oetker, Obergösgen, Germany) was prepared according to the manufacturer’s instruction. Following dissolution of the gelatin leafs, a drop of red ink (Waterman, Paris, France) was added to the preparation. It was applied to the glass slide while still warm to establish a first uniform thick layer of 500 µm (±200 µm). A sheet of cigarette paper was then applied onto the gelatin surface, careful to prevent formation of wrinkles or air pockets. A second layer of gelatin was then applied over the paper aiming at a thickness of 250 µm (±100 µm). Each preparation was then cooled for at least 5 min at 5 °C. A schematic of the model is provided in Fig. 1.

### Experimental set-up

**Fig. 2:**
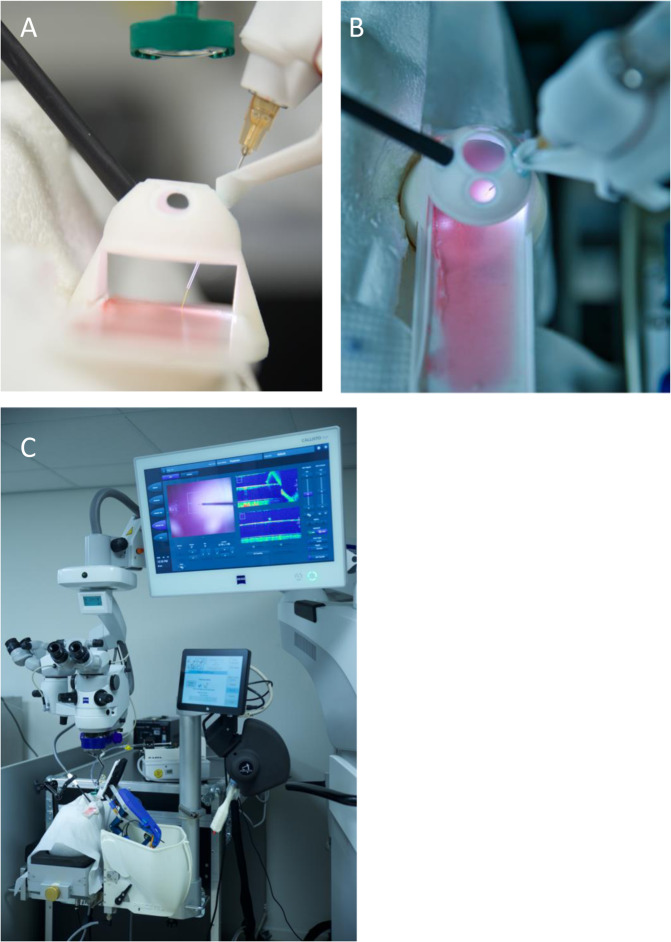
Photographic representation of the experimental surgical setup. **a** A lateral view of the model eye containing the glass slide. Both the needle and the illumination are inserted through openings in the plastic shell. The set-up is held by a clamp and positioned under an OPMI Lumera 700 surgical microscope. **b** A vertical view of the model eye. **c** The disposition of the model eye relative to the microscope and the PSS robotic arm and motion controller.

The microscope slide containing the model retina was mounted in a plastic holder specifically designed to hold the slide firmly in a horizontal position while providing at an appropriate distance above the slide, a spherical frame that simulated the anterior sclera. Appropriate openings were present in the sphere to simulate the edge of the limbus, and sclerotomy sites required to illuminate the slide, and for the passage of an injection needle (Fig. 2a). The model was set-up on a dummy head and positioned under an OPMI LUMERA 700 surgical microscope fitted with a Resight visualization system (Carl Zeiss Meditec AG, Jena, Germany) as seen in Fig. 2b. The intraoperative OCT (iOCT) system was optimally positioned to visualize the tip of the needle as it reached the surface of the gelatin, allowing the surgeon during the injection procedure to view the screen as needed. The monitor was positioned at an appropriate angle for the surgeon to easily switch from the microscope to the screen with the OCT. Surgeons were seated on a comfortable surgical chair fitted with arm rests.

A 38G outer diameter polyamide needle 5 mm in length (model 3233, MedOne Surgical Inc, Sarasota, FL, USA), was used for the injection. It was fixed to a PVC extension tube (model 3243, MedOne Surgical Inc, Sarasota, FL, USA) connected to a standard PVC connection tubing (model 5011973, B.Braun, Melsungen, Germany) while the proximal end of the tubing was connected to a 1 mL tuberculin syringe mounted to an infusion pump (Model R99-E Razel Scientific Instruments, Saint Albans, VT, USA). The pump was set at an infusion rate of 10 µL/min. A mixture made of 5 droplets of green ink (Waterman, Paris, France) and 100 ml of water was used as an infusate. Along the edge of the polyamide needle, a fiberoptic cable of 300 µm outer diameter was secured. The proximal fiber was connected to a spectral-domain OCT engine (Ganymede-II-SP12; Thorlabs, Germany) with a light source centered at 930 nm. This allowed the acquisition of A-scan OCT (OCTa) distance measurements on a continuous basis at a frequency of 25kHz. The reference point used for these measurements was the end-surface of the OCT fiber.

The precision micromanipulator used in these experiments was a Preceyes surgical system model R0 (Preceyes BV, Eindhoven, the Netherlands). PSS is a telemanipulation robotic system that is characterized by two physical distinct parts interacting with each other in a master-slave configuration via a computer [20].

The motion controller is positioned adjacent to the microscope in easy reach of the surgeon’s right hand. Via the dedicated computer, the instrument manipulator within the model eye is controlled to perform tasks under the surgeon’s control. The positioning of PSS in these experiments can be seen in Fig. 2b.

### Participants and experimental protocol

During the Euretina 2019 congress in Paris France, vitreoretinal surgeons were invited to participate in a trial on subretinal delivery comparing manual surgery with the PSS at the Preceyes booth within the commercial area. This study did not involve any therapeutic action, and for this reason did not require an ethics committee approval according to the Central Committee on Research Involving Human Subjects (CCMO), The Hague, the Netherlands. However, all participants did sign a consent indicating that they agreed to have the experiment recorded for subsequent analysis and publication.

Surgeons were given a 5 minutes description of the experimental set-up and instructed on the use of the PSS. Following a description and intent of the study, they were allowed to practice with the robotic system for about 5 minutes. Participants were randomized to start with either a manual approach or using the PSS. The following sequence of events was required in each arm of the study: (1) advance the needle to the gelatin surface, (2) pierce the gelatin to an appropriate depth (contact of the needle with the cigarette paper) using as a guide the iOCT and/or the landmarks provided by the model as visualized through the microscope, (3) instruct the assistant to start the injection pump, (4) maintain the tip of the cannula in this location until a bleb was visible under the upper gelatin or for the length of the injection (maximum injection time set at 60 s).

The bleb was seen as an elevation of the upper gelatin as visualized through the microscope and/or the iOCT. Reflux was noted as a pooling of fluid on the surface of the gelatin.

For each attempt, the following data was collected and stored for post analysis: a video recording using the camera on the Zeiss OPMI Lumera 700; the OCT A distance sensor measurements; the iOCT recordings.

### Data analysis

All recordings were reviewed with regards to the following parameters: (1) success in creating a bleb; (2) presence of reflux; (3) injection duration; (4) drift; (5) tremor; (6) increase in the diameter of the puncture hole. The definition for each parameter is provided in Table 1.

**Table 1:** Definition of parameters of surgical success used in the assessment of the experiments.

Surgical outcome	Defined as
Bleb creation	Presence of injected solution under the upper gelatin layer as observed on intraoperative OCT and/or microscope recordings (visual observation).
Reflux	Appearance of solution above the upper gelatin layer observed on intraoperative OCT and/or microscope recordings (visual observation).
Injection duration	Time in seconds from start to stop of the injection pump.
Time in seconds from start to stop of the injection pump	Amplitude of movements of the injection cannula extremity over a short time of 0.02 second (μm) in the Z axis.
Hole enlargement	Presence of a noncircular hole in the upper gelatin layer observed on intraoperative OCT and/or microscope recordings after retraction of the instrument.

## Results

Nine vitreoretinal surgeons participated in the study and completed both manual and robotic-assisted injections. There were eight men ranging from beginner [2] to experienced [6] and one experienced woman. Experience was defined as having performed more than 200 vitreoretinal procedures as compared to less. One surgeon (male, beginner) had previously used the robotic system. We did not observe a greater ability of younger surgeons to perform the task compared to older surgeons.

In three cases, the recording from the OCTa device were excluded due to technical issues and were eliminated from the analysis of drift and tremor.

In the manually performed injections, a bleb was created in 4/9 attempts. These were associated with reflux at the surface in 8/9 cases. The reflux started during the injection in 7/9 cases and in 1/9 at the appearance of the bleb. With PSS assistance, a bleb was created in this model in 8/9 attempts, and reflux was observed in 7/9 cases, starting before or during bleb initiation.

**Fig. 3:**
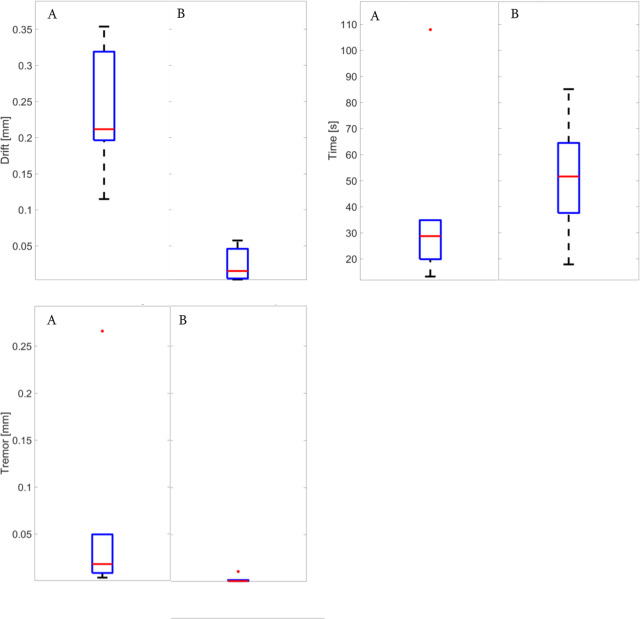
Quantification of injection parameters. Boxplot comparisons of drift, tremor, and injection time between the manual approach (**a**) and robotic assistance (**b**). The red line represents the mean value, the blue box represents the upper and lower quartiles, the black lines outside the box extend to the highest and lowest observations and the red dot corresponds to outlier value.

Enlargement of the puncture hole was observed in 7/9 of manual attempts and was absent with PSS assistance, despite the fact that injections with PSS were of longer duration. For manual injections, the median time was 29 s (range 13–108 s), while it was 52 s (range 18–85 s) with PSS assistance. The ability to hold the needle tip steady (tremor) was significantly improved by PSS assistance, with a median variation in position of 1 µm (range 1–11 µm) as compared to manually performed injections where a median of 18 μm (range of 4–266 μm) was observed. Similarly, avoiding a drift away from the initial position during the period of injection, was improved with the PSS. With PSS assistance, the median drift was 16 μm (range 4–58 µm) while in manual attempts, the median drift of 212 µm (range 115–355 µm). Figure 3 summarizes these findings. In all cases, PSS led to a stabilization of both parameters for the duration of the experiment. In the manual mode, some surgeon showed a significant ability to control both parameters, while others had less control. The presence of tremor was usually associated with more drift.

## Discussion

Performing a SI of gene vectors can be surgically challenging, particularly in attached retina. Using a telemanipulation robotic system may facilitate the procedure, as it assists the surgeon by providing increased precision and stability. It reduces tremor and is able to maintain a fixed position for a prolonged if not indefinite period of time [21]. Key to the successful completion of the procedure are the ability to reach and maintain the needle tip in the appropriate location for a sufficient period of time. Once in position, there should be no tendency to move away from the location (drift), nor should there be any significant vibration (tremor) in XYZ. The aim of the current paper was to evaluate these positional characteristics in both manual and assisted tasks, and determine which robotic characteristics contributed most.

Evaluating surgical tasks is subject to many variables. These can be minimized by using an artificial set-up, which centers around the parameters of interest while limiting all other variables. To this end, we used a state-of-the-art microscope with integrated iOCT, a mock retinal model. The set-up simulated the operative setting while the gelatin model simplified the retina to the key components of interest.

Using the experimental setup, it was possible to show that when performed manually, the ability to create a bleb and its size was highly variable from surgeon to surgeon, while PSS improved the overall ability to generate a bleb. Reflux was present in both modes, but this may be reflective of the lack of elastic structures in the model as this was not observed with fresh cadaveric porcine eyes or in live animals [22]. However, the lack of elastic properties allowed us to assess variations in puncture hole size, a correlate of tip lateral movement during the injection. Present in 7/9 manual procedures, such an enlargement was absent using PSS.

Injections with PSS were twice as long on average as compared to manual despite the fact that other parameters such as the rate and maximum time of injection were identical in both cases. There was also no correlation with the ability to form a bleb. Tanaka et al compared robotic and manual injection in a vein cannulation model [23]. They also found that injection times with assistance were twice as long. Motion stability was particularly important as it allowed the surgeon to remain within the vein for a longer period of time as it canceled out tremor. The subretinal space may present a similar challenge. The ability to remain comfortably in the subretinal space allows for a slow controlled subretinal infusion allowing for the complete delivery of a target volume. This has been identified as a crucial element to the success of subretinal gene/cell therapy [16, 24].

In addition to reducing tremor, our experimental system reduced drift. Both were reduced by a factor of 10 or more as compared to manual delivery. While physiological tremor is estimated at a frequency of 8–12 Hz, drift, which is an additional low-frequency involuntary component of human hand movement below 3 Hz [25] has a crucial limiting impact on the surgeon’s ability to perform a static task. Cancellation of drift compared to tremor is more complicated due to its imbrication with voluntary movements [25]. Telemanipulation robots such a PSS are able to suppress both tremor and drift making them particularly well suited for subretinal delivery.

The combination of iOCT and PSS was considered helpful by most surgeons. It provided real-time visual feedback while the probe was being positioned. De Smet et al. showed that depth perception through a microscope is limited and decreases the surgeon’s ability to perform precision tasks [13]. The iOCT provided the required resolution, but due to its highly magnified nature, it is best used when the surgeon does not need to concentrate on the XY position of the probe. However, to make optimal use of the iOCT, certain additional factors need to be taken into account. In comparing the video recordings obtained in the course of these experiments with the iOCT recordings, there were cases where the video image more clearly identified the onset of the bleb than the iOCT. In some cases, the needle blocked visualization of the subretinal space (the iOCT must be centered at the tip of the catheter or just beyond), in other cases, automatic averaging of the OCT signaled to an interruption in the visualization of the structure within the gelatin layer. Reducing or shutting off the OCT averaging mode and appropriate calibration of the iOCT prior to the procedure are important to avoid these problems.

In conclusion, we describe an artificial retina model that simulates SI and its constraints in order to compare and quantify the differences between vitreoretinal surgeons performing the procedure with and without robotic assistance. Robotic assistance enhances motion stability, suppresses time constraint resulting in an improvement of surgical performance for all the participants, and in standardization of the technique. Greater improvement was noted in surgeons who were less successful manually. Reduced enlargement of the surgical hole and increased injection time will also translate in less reflux of gene product in a retinal tissue with normal elastic tissue.
